# ADH-Enhancer: an attention-based deep hybrid framework for enhancer identification and strength prediction

**DOI:** 10.1093/bib/bbae030

**Published:** 2024-02-22

**Authors:** Faiza Mehmood, Shazia Arshad, Muhammad Shoaib

**Affiliations:** Department of Computer Science, University of Engineering and Technology Lahore, (Faisalabad Campus) Pakistan; Department of Computer Science, University of Engineering and Technology Lahore, 54890, Pakistan; Department of Computer Science, University of Engineering and Technology Lahore, 54890, Pakistan

**Keywords:** enhancer identification, enhancer strength prediction, transfer learning, attention mechanism, convolutional neural network

## Abstract

Enhancers play an important role in the process of gene expression regulation. In DNA sequence abundance or absence of enhancers and irregularities in the strength of enhancers affects gene expression process that leads to the initiation and propagation of diverse types of genetic diseases such as hemophilia, bladder cancer, diabetes and congenital disorders. Enhancer identification and strength prediction through experimental approaches is expensive, time-consuming and error-prone. To accelerate and expedite the research related to enhancers identification and strength prediction, around 19 computational frameworks have been proposed. These frameworks used machine and deep learning methods that take raw DNA sequences and predict enhancer’s presence and strength. However, these frameworks still lack in performance and are not useful in real time analysis. This paper presents a novel deep learning framework that uses language modeling strategies for transforming DNA sequences into statistical feature space. It applies transfer learning by training a language model in an unsupervised fashion by predicting a group of nucleotides also known as k-mers based on the context of existing k-mers in a sequence. At the classification stage, it presents a novel classifier that reaps the benefits of two different architectures: convolutional neural network and attention mechanism. The proposed framework is evaluated over the enhancer identification benchmark dataset where it outperforms the existing best-performing framework by 5%, and 9% in terms of accuracy and MCC. Similarly, when evaluated over the enhancer strength prediction benchmark dataset, it outperforms the existing best-performing framework by 4%, and 7% in terms of accuracy and MCC.

## INTRODUCTION

Deoxyribonucleic acid (DNA) contains diverse types of information about living organisms from their birth till death [1]. Based on physico-chemical properties and roles in performing biological functions, DNA sequence is categorized into two main regions: coding and noncoding. Coding region contains genes that get expressed and produce proteins, while noncoding region affects the production of proteins [2]. Furthermore, which genes need to be expressed at what point in time is controlled by specific genes and regulatory elements present in the noncoding regions of DNA [3]. Regulatory elements of noncoding region including promoters, enhancers, silencers and insulators regulate the process of converting coding DNA into messenger ribonucleic acid (RNA) (transcription) that is later translated into proteins which are essential for the development, growth and functioning of organisms [3–5].

Among the different types of regulatory elements, enhancers are considered more important to control expression rate of genes [3]. To exploit the destiny of cells during evolution and differentiation, enhancers bio-molecules enhance the transcription of targeted genes. These bio-molecules facilitate a platform to transcription factors (TFs) that recruit diverse co-activators including RNA polymerase II initiation as well as elongation factors. Enhancers posses different strength; the stronger the enhancer is, the longer it can support TFs and other regulatory machinery to execute transcription. Irregularities in the presence of enhancers and their strength, TFs and co-activators lead to the development of various diseases and disorders [6, 7] such as Cancer [8]. Moreover, it has been discovered that genetic mutations within enhancers can cause severe illness including neurodegenerative disease [9–12], bladder cancer [13], Hirschsprung disease [14] and inflammatory bowel disease [15].

Considering the impact of enhancers and their strength in diverse types of biological processes and to explore their additional roles in diseases propagation [3], development of diverse types of methodologies for accurate identification of enhancers and their strength is an active area of research in Genomics sequence analysis [2, 16]. Enhancers are being identified using various methods such as genome-wide correlation with chromatin data, conservation analysis, transcription of a reporter gene and enhancer RNA (eRNA) measurement. The complex of DNA and proteins that make up chromosomes is called chromatin. In genome-wide correlation with chromatin data analysis, scientists study the interaction between DNA and chromatin-associated proteins in order to identify the regions of the genome that are likely to be enhancers. This correlation helps to narrow down the potential enhancer regions. Particularly, genomic regions exhibiting enhancer activity are often marked by chromatin enrichment in H3K4me1, H3K27ac and the chromatin modifier p300 histone acetyltransferase, serving as genome-wide markers [17, 18]. Since enhancers use TFs and associated cofactors necessary for their activation, they are nucleosome-deficient. Candidate enhancers are often identified by assessing accessible chromatin regions away from transcriptional start sites (TSSs), a process inferred through techniques such as DNase-seq and ATAC-seq [19, 20]. However, these approaches rely on certain genetic or biological features such as occupation of TFs, hence they only manage to detect certain portion of enhancers because not all enhancers are occupied by the TFs [21]. Furthermore, these approaches are labor-intensive, time-consuming and expensive [21].

Evolutionary conservation analysis also plays a crucial role in identifying enhancers. If a DNA sequence is conserved across different species, it suggests that the sequence has been preserved over time because of its functional importance. By comparing the genomes of different species, researchers have been identifying regions that are highly conserved and likely to be enhancers. However, it is important to note that many enhancers cannot be identified only through sequence conservation [22, 23]. Furthermore, another method being used for enhancer identification involves the transcription of a reporter gene. A reporter gene is a gene that can be easily observed or measured, and its expression is used as an indicator of enhancer activity. Scientists insert the reporter gene into a DNA construct along with the suspected enhancer sequence. If the enhancer is active, it will bind to TFs and increase the expression of the reporter gene. This increased expression can be easily detected and measured. Enhancers are also being identified through eRNA measurement. eRNAs are short noncoding RNA molecules that are transcribed from active enhancer regions. By measuring the levels of eRNAs, scientists can infer the activity of enhancers. eRNAs are bidirectional transcription that occurs from enhancer TSSs, often coinciding with established enhancer histone marks. eRNAs offer high specificity in detecting enhancers compared with histone modifications due to their single-base resolution in nascent transcripts [24, 25]. These eRNAs are identified through analyses like CAGE, enriching for active 5’ TSSs, or nascent transcript analyses like PRO-seq and GRO-seq, where transcript expression levels quantify enhancer activity functionally [24, 26–28]. These genome-wide approaches have revealed millions of enhancer candidates across different cell types and tissues in metazoans. Nonetheless, confirming the authenticity of these candidates remains a major obstacle.

With an aim to replace experimental approaches with computational methods, taking into account the wide success of artificial intelligence (AI) approaches in diverse Genomics and Proteomics sequence analysis tasks [29, 30], several AI-supported enhancers identification and strength prediction frameworks have been proposed [2, 31–37]. Working paradigm of these frameworks can be categorized into two different modules. The first module transforms raw DNA sequences into statistical feature space and the second module, which comprises of machine or deep learning classifier, discriminates enhancers from non-enhancers and also differentiates weak enhancers from strong enhancers. Initially the focus of researchers was to develop computational frameworks that take only raw DNA sequences and identy the presence or absence of enhancers [38–42]. With this particular motivation five different computational frameworks were developed: CSI-ANN [38], RFECS [41], EnhancerFinder [39], EnhancerDBN [40] and BiRen [42]. In 2016, Liu *et al*. [33] developed the very first framework namely iEnhancer-2L [33] that distinguishes enhancers from other regulatory elements and determines the strength of enhancers. iEnhancer-2L [33] leveraged the pseudo k-tuple nucleotide composition (PseKNC) encoder to characterize DNA sequences into statistical vectors. In the following years, to further improve the performance, researchers have developed three different frameworks: EnhancerPred [31], EnhancerPred 2.0 [32] and iEnhancer-PsedeKNC [33]. These frameworks used physico-chemical properties for the transformation of DNA sequences into statistical vectors. Furthermore, eight existing frameworks, namely, iEnhancer-EL [43], iEnhancer-2L [33], tan2019ensemble [44], iEnhancer-XG [45], iEnhancer-MFGBDT [34], iEnhancer-KL [46], iEnhancer-RD [36], iEnhancer-Deep [16], and Enhancer-FRL [35] transform DNA sequences into statistical feature space by combining the power of different sequence encoding methods including one-hot encoding, k-mer frequency, nucleotide composition, physico-chemical properties and sub-sequence profiles. Moreover, three frameworks [47–49] utilize Bidirectional Encoder Representations from Transformers (BERT) representations.

With an aim to transform DNA sequences into statistical vectors by capturing both discriminative and semantical relationships of nucleotides, researchers developed two frameworks, iEnhancer-5Step [37] and Enhancer-DSNet [2], that used unsupervised and supervised neural k-mer embedding generation approaches.

At the classification stage, among the 19 existing frameworks, nine frameworks [12, 31–33, 37, 43, 45, 50] used machine learning classifiers including support vector machine (SVM), Logistic regression, XGBoost, Random forest, K-neighbors and Naive bayes. Four frameworks [16, 47, 48] use convolutional neural network (CNN) based classifiers, two frameworks [44, 51] use hybrid model combination of CNN and Long Short-Term Memory (LSTM) based classifier, one framework [52] utilizes the effect of attention along with hybrid network (CNN-LSTM) and one framework only uses the linear layer as classifier. Furthermore, two frameworks rely on generative adversarial network (GAN) [36, 51] based classifiers and the most recent framework [53] uses the Laplacian regularized radial function based classifier for enhancer identification and strength prediction tasks.

The top-performing enhancer identification and strength prediction approaches utilize word embedding approaches (DNA2Vec [54], SuperDNA2Vec [1]) and language model BERT [49] [48] to better characterize enhancer sequences. However, DNA2Vec [54] fails in capturing comprehensive residue relations with target classes and SuperDNA2Vec [1] fails in capturing comprehensive long-range residue-to-residue relations and translational in-variances of residues. Bert language model training mechanism comprises of two different strategies namely masked words prediction and next sentence prediction. In natural language processing (NLP) it has produced state-of-the-art performance in diverse types of classification tasks including document classification, sentiment analysis and fake news detection [55?? –57]. Unlike NLP tasks in biological sequence analysis BERT remains a failure in extracting neucleotides semantics and discriminative potential because here the next sentence prediction is not possible and the model is trained only based on masked k-mers.

A critical analysis of the existing frameworks reveals that although these frameworks have achieved reasonable performance figures for enhancer identification task, they still lack in precisely identifying the strength of enhancers. Among the five most recent frameworks, iEnhancer-MRBF [53] and iEnhancer-DLRA [58] achieve top accuracy of almost 80% for enhancer identification task and around 76% for enhancer strength prediction task. Considering the room for performance improvement, contributions of this paper are manifold:

- With an aim to generate comprehensive statistical feature space of raw sequences the proposed framework reaps the benefit of Average Stochastic Gradient Descent–Long Short-Term Memory (AWD-LSTM) based ULMFIT language model; we trained ULMFIT in an unsupervised fashion to learn the distribution of neucleotides in DNA sequences.
- It presents a novel CNN and self-attention-based classifier that facilitates to learn and extract informative patterns of neucleotides in a more precise and comprehensive manner.
- The proposed framework is evaluated over two benchmark datasets of enhancer identification and strength prediction under two different experimental settings, i.e. 5-fold cross-validation and independent test sets.
- It explores performance impact of different sizes k-mers for generating informative and more discriminative features among sequences of distinct classes.
- The proposed framework outperforms the existing enhancer identification and strength prediction frameworks over two public benchmark datasets in terms of four distinct evaluation measures.

## RELATED WORK

In the marathon of developing robust and precise AI framework for enhancers identification and their strength prediction, to the best of our knowledge, 19 different frameworks have been proposed, which are summarized in this section.

Liu *et al*. [33] presented the very first framework, iEnhancer-2L, that discriminates enhancers from other regulatory elements and also estimates their strength. iEnhancer-2L leveraged the PseKNC encoder for transforming raw DNA sequences into statistical vectors and SVM classifier for discriminating enhancers from non-enhancers and strong enhancers from weak enhancers. The authors performed experimentation over enhancers identification and strength prediction core datasets and independent test sets to explore the effectiveness of the proposed iEnhancer-2L [33] framework. On core datasets of enhancers identification and strength prediction, iEnhancer-2L managed to produce 76.89% and 61.93% accuracy, 78.09% and 62.21% sensitivity, 75.88% and 61.82% specificity and 0.54 and 0.24 MCC, respectively. Similarly on independent test sets it produced 73% and 60.5% accuracy, 71% and 47% sensitivity, 75% and 74% specificity and 0.46 and 0.218 MCC, respectively. To improve the predictive performance of the iEnhancer-2L [33] framework, Jia *et al*. [31] developed EnhancerPred that transforms raw DNA sequences into statistical feature space by utilizing three different sequence encoding methods: Bi-profile Bayes, nucleotide composition and pseudo-nucleotide composition. Generated feature space along with SVM classifier managed to produce 73.18% and 62.06% accuracy, 72.57% and 62.67% sensitivity, 73.79% and 61.46% specificity and 0.43 and 0.24 MCC, over core datasets of enhancers identification and strength prediction, respectively. Similarly on independent test sets of enhancers identification and strength prediction it produced 74% and 55% accuracy, 73.5% and 45% sensitivity, 74.5% and 65% specificity and 0.48 and 0.102 MCC, respectively.

**Table 1:** A Summary of Feature Encoding Schemes, Experimental Datasets and Computational Approaches Proposed For Enhancer Identification and Strength Prediction Task

**Author**	**Feature Encoding**	**Classifier**	**Dataset**	**Performance**
Liu *et al*-2016 [33]	PseKNC	SVM	Benchmark Dataset, & Independent Test set	Layer-1 SN=78.09, SP=75.88, ACC= 76.89, MCC= 0.54, AU-ROC=0.85 Layer-2 SN=62.21, SP=61.82, ACC= 61.93, MCC=0.24, AU-ROC 0.66 & Layer-1 SN=71.0 SP=75.0 ACC=73.00 MCC=0.460 AU-ROC=80.62 Layer-2 SN=47.00, SP=74.00, ACC= 60.50, MCC= 0.218, AU-ROC= 66.78
Jia *et al*-2016 [31]	Bi-profile Bayes+ Nucleotide composition+ pseudo-nucleotide composition	SVM	Benchmark Dataset & Independent Test set	Layer-1 SN=71.97 SP=82.82 ACC=77.39, MCC= 0.55, AU-ROC=N/A Layer-2 SN=71.16, SP=65.23, ACC=68.19, MCC=0.36 & Layer-1 SN=73.5 SP=74.5 ACC=74.00 MCC=0.480 AU-ROC=80.13 Layer-2 SN=45.00, SP=65.00, ACC= 55.00 MCC=0.102 AU-ROC=57.90
Liu *et al*-2016 [59]	Pseudo degenerate kmer nucleotide composition	SVM	Benchmark Dataset	Layer-1 SN=77.31, SP=76.30, ACC=76.78, MCC=0.54, AU-ROC=0.85 Layer-2 SN=62.62, SP=64.41, ACC=63.41, MCC=0.27, AU-ROC=0.69
He *et al*-2017 [32]	position-specific trinucleotide propensity + EIIP of trinucleotides+ F-score feature selection	SVM	Benchmark dataset	Layer-1 SN=87.94, SP= 88.61, ACC= 88.27, MCC= 0.77 Layer-2 SN=97.98, SP= 98.11, ACC= 98.05, MCC= 0.96
Bin Liu *et al*-2018 [43]	kmer+subsequence profile +PseKNC	SVM	Benchmark Dataset & Independent test set	Layer-1 SN=75.67, SP= 80.39, ACC=78.03 MCC=0.5613, AU-ROC=85.47 Layer-2 SN=69.00, SP=61.05, ACC= 65.03, MCC= 0.3149AU-ROC= 69.57 & Layer-1 SN=71.0, SP=78.5, ACC=74.75, MCC=0.496, AU-ROC=81.73 Layer-2 SN=54.00,SP= 68.00 ACC=61.00 MCC=0.222 AU-ROC=68.01
Tan *et al*-2019 [44]	One hot encoding + physicochemical properties	CNN+RNN Ensemble	Benchmark dataset & Independent Test set	Layer-1 SN=73.25, SP= 76.42, ACC= 74.83, MCC= 0.498, AU-ROC= 76.94 Layer-2 SN=58.96, SP= 38.28, ACC= 79.65, MCC= 0.197, AU-ROC= 60.68 & Layer-1 SN= 75.5, SP= 76.00, ACC= 75.50, MCC= 0.51 AU-ROC= 77.04 Layer-2 SN= 83.15, SP= 45.61, ACC= 68.49, MCC= 0.312AU-ROC= 67.14
Le *et al*-2019 [37]	Neural word embeddings	SVM	Benchmark dataset & Independent Test set	Layer-1 SN=81.1, SP=83.5, ACC=82.3, MCC=0.65 Layer-2 SN=75.3, SP=60.8, ACC=68.1, MCC=0.37 & Layer-1 SN=82, SP=76, ACC=79, MCC=0.58 Layer-2 SN=74, SP=53, ACC=63.5, MCC=0.28
Asim *et al*-2020 [2]	K-mer representaion by fusing k-mer positional information with sequence type	Precise Softmax Classifier	Benchmark dataset & Independent Test set	Layer-1 SN=76.0, SP=76.0, ACC=76.0, MCC=0.52 Layer-2 SN=67.0, SP=67.0, ACC=63.0, MCC=0.26 & Layer-1 SN=78.0, SP=77, ACC=78, MCC=0.56 Layer-2 SN=83.0, SP=67.0, ACC=83.0, MCC=0.70
Le *et al*-2021 [48]	BERT Embeddings	CNN	Benchmark dataset & Independent Test set	Layer-1 SN=79.5, SP= 73, ACC=76.2, MCC=0.525 & Layer-1 SN=80, SP=71.2, ACC=75.6, MCC=0.514
Lim *et al*-2021 [60]	binary +debinary +ANF +NCP + ENAC, KGAP	RF	Benchmark dataset & Independent Test set	Layer-1 SN=73.64, SP= 78.71, ACC=76.18, MCC=0.5264, AUC=84 Layer-2 SN=68.46, SP=56.61, ACC=62.53, MCC=0.2529, AUC=67 & Layer-1 SN=78.50, SP=81, ACC=79.75, MCC=0.5952 Layer-2 SN=93, SP=77.0, ACC=85, MCC=0.7091
Cai *et al*-2021 [45]	Mismatch k-tuple +PSSM +Spectrum +Subsequence Profile + PseDNC	XGBoost	Benchmark dataset & Independent Test set	Layer-1 SN=75.7, SP= 86.5, ACC=58.55, MCC=0.62 Layer-2 SN=74.94, SP=58.55, ACC=66.74, MCC=0.33 & Layer-1 SN=74.0, SP=77.5, ACC=75.75, MCC=0.514 Layer-2 SN=70.0, SP=57.0, ACC=63.5, MCC=0.272
Kamran *et al*-2022 [16]	One hot encoding	CNN	Benchmark dataset & Independent Test set	Layer-1 SN=86.99, SP=88.54, ACC=87.77, MCC=0.75 Layer-2 SN=83.57, SP=78.16, ACC=80.86, MCC=0.62 & Layer-1 SN=81.5, SP=67, ACC=74.02, MCC=0.4902 Layer-2 SN=73.0, SP=49.0, ACC=61, MCC=0.226
Yang *et al*-2021 [36]	Skip-gram	GAN	Independent Test set	Layer-1 SN=81.1, SP=75.8, ACC=78.4, MCC=0.567 Layer-2 SN=96.1, SP=53.7, ACC=74.9, MCC=0.505
Liao *et al*-2022 [52]	Word2vec	CNN-LSTM-Attention	Benchmark dataset & Independent Test set	Layer-1 SN=84.18, SP=82.45, ACC=83.32, MCC=0.666 Layer-2 SN=89.27, SP=77.33, ACC=83.3, MCC=0.673 & Layer-1 SN=78, SP=78.50, ACC=78.25, MCC=0.565 Layer-2 SN=87, SP=69.0, ACC=78, MCC=0.569
Luo *et al*-2022 [47]	DNABERT	CNN	Benchmark dataset & Independent Test set	Layer-1 ACC=79.4, MCC=0.593, AUC= 87.9 Layer-2 ACC=65.3, MCC=0.31, AUC=70.3 & Layer-1 ACC=79.3, MCC=0.585, AUC= 84.4 Layer-2 ACC=70.1, MCC=0.401, AUC= 81.2
Geng *et al*-2022 [51]	GAN + FastText	LSTM-CNN	Benchmark dataset & Independent Test set	Layer-1 SN=74.87, SP=75.63, ACC=75.25, MCC=0.5051 Layer-2 SN=70.68, SP=68.89, ACC=69.7, MCC=0.3954 & Layer-1 SN=74.87, SP=75.63, ACC=75.25, MCC=0.5051 Layer-2 SN=70.68, SP=68.89, ACC=69.7, MCC=0.3954
Xiao *et al*-2023 [58]	kmer + ANF + NBP	RBF	Benchmark dataset & Independent Test set	Layer-1 SN=79.52, SP=82.95, ACC=81.23, MCC=0.6254, AUC= 88.09 Layer-2 SN=77.23, SP=79.69, ACC=76.95, MCC=0.5419, AUC=84.09 & Layer-1 SN=82, SP=77.50, ACC=79.75, MCC=0.5956 Layer-2 SN=100, SP=67.0, ACC=83.5, MCC=0.7098
Li *et al*-2023 [49]	BERT	Linear	Benchmark dataset	Layer-1 SN=93.73, SP=95.75, ACC=94.74, MCC=0.8951 Layer-2 SN=80, SP=86, ACC=83, MCC=0.6612

Liu *et al*. [59] developed another computational framework namely iEnhancer-PsedoKNC that generates sequence representation using pseudo degenerate kmer nucleotide composition encoding scheme and performed classification using SVM classifier. Experimentation on benchmark datasets indicates the dominance of iEnhancer-PsedeKNC compared with iEnhancer-2L [33] and EnhancerPred [31]. Over core datasets, the iEnhancer-PsedoKNC framework produced 76.78% and 63.41% accuracy, 77.31% and 62.62% sensitivity, 76.30% and 64.41% specificityand 0.54 and 0.27 MCC, respectively. He *et al*. [32] developed EnhancerPred2.0 that transforms DNA sequences into statistical vectors using physio-chemical property based encoding method namely electron–ion interaction potential (EIIP) encoder with position-specific tri-nucleotide propensity. Furthermore, it removes irrelevant and redundant features through wrapper-based feature selection method. SVM classifier along with selected features produced 88.27% and 98.05% accuracy, 87.94% and 97.98% sensitivity, 88.61% and 98.11% specificity and 0.77 and 0.96 MCC, over core datasets of enhancers identification and strength prediction, respectively.

Liu *et al*. [43] presented iEnhancer-EL, which transforms raw DNA sequences into statistical feature space using three different sequence encoding methods including NAC, subsequence profile and PseKNC. At the classification stage, enhancer sequences discrimination from non-enhancer sequences, the authors proposed a meta predictor that generates probabilistic feature space of SVM-based six different encoders, namely, PseKNC-77, PseKNC-81, PseKNC-4113, subsequence-profile-64, kmer-64 and kmer-4096 using SVM classifier. Similarly, for weak enhancers discrimination from strong enhancers, the authors developed a meta predictor that generates probabilistic feature space of SVM-based 10 different encoders, namely, PseKNC-9, PseKNC-9, PseKNC-9, PseKNC-13, PseKNC-29, PseKNC-77, PseKNC-81, PseKNC-265, kmer-64 and kmer-4096. On core datasets of enhancers identification and strength prediction, it produced 78.03% and 65.03% accuracy, 75.67% and 69.00% sensitivity, 80.39% and 61.05% specificity and 0.56 and 0.31 MCC, respectively. Similarly on independent test set of enhancers identification and strength prediction datasets it produced 74.75% and 61% accuracy, 71% and 54% sensitivity, 78.5% and 68% specificity and 0.49 and 0.222 MCC, respectively.

Tan *et al*. [44] proposed an ensemble of CNN and recurrent neural network for efficient identification of enhancers and their strength. For sequence representation, they employed six different kinds of physico-chemical properties, namely, Rise, Roll, Shift, Slide, Tilt and Twist. Performance comparison with existing frameworks using benchmark datasets and independent test sets indicated that the proposed framework outperforms the existing three computational frameworks, iEnhancer-EL [43], iEnhancer-2L [33] and EnhancerPred [31], with decent margin across different evaluation metrics. On core datasets of enhancers identification and strength prediction, it produced 74.83% and 79.65% accuracy, 73.25% and 58.96% sensitivity, 76.42% and 38.28% specificity and 0.498 and 0.197 MCC, respectively. Similarly on independent test sets of enhancers identification and strength prediction datasets it produced 75.5% and 68.49% accuracy, 75.5% and 83.15% sensitivity, 76% and 45.61% specificity and 0.51 and 0.312 MCC, respectively. Le *et al*. [37] presented the iEnhancer-5Step framework that utilizes neural k-mer embeddings based on sub-word information to effectively identify enhancers and their strength along with SVM classifier. On core datasets of enhancers identification and strength prediction, iEnhancer-5Step produced 82.3% and 68.1% accuracy, 81.1% and 75.3% sensitivity, 83.5% and 60.8% specificity and 0.65 and 0.37 MCC, respectively. Similarly on independent test sets of enhancers identification and strength prediction it produced 79% and 63.5% accuracy, 82% and 74% sensitivity, 76% and 53% specificity and 0.58 and 0.28 MCC, respectively.

Asim *et al*. [2] proposed Enhancer-DSNet that captures position as well as semantics of residues; they generated sequence representation by fusing k-mer position and sequence type information. A rich performance comparison with the existing computational frameworks proved that Enhancer-DSNet outperformed all frameworks across both core dataset and independent test in terms of distinct evaluation metrics. On benchmark core datasets of enhancers identification and strength prediction, it produced 76% and 63% accuracy, 76% and 67% sensitivity, 76% and 67% specificity and 0.52 and 0.26 MCC, respectively. Similarly on independent test sets of enhancers identification and strength prediction datasets it produced 78% and 83% accuracy, 78% and 83% sensitivity, 77% and 67% specificity and 0.56 and 0.70 MCC, respectively. Cai *et al*. [45] proposed the iEnhancer-XG framework that discretizes the DNA sequences ensembling of five different sequence encoding methods, namely, Mismatch k-tuple, Position-specific scoring matrix (PSSM), Spectrum, Subsequence Profile and Pseudo dinucleotide composition (PseDNC) and performed classification using the XGBoost classifier. The authors performed experimentation using 10-fold cross-validation on core benchmark datasets and independent test set of enhancers identification and their strength prediction. On core enhancers identification benchmark dataset they achieved 58.55%, 75.7%, 86.5% and 0.62 in terms of accuracy, sensitivity, specificity and MCC, respectively. On independent test sets of enhancers identification and strength prediction datasets they achieved 75.75% and 63.5% accuracy, 74% and 70% sensitivity, 77.5% and 57% specificity and 0.514 and 0.272 MCC, respectively.

Lim *et al*. [60] proposed iEnhancer-RF framework by utilizing six different types of sequence encoding methods, namely, binary, dibinary, NCP, XY KGAP, ENAC and accumulated nucleotide frequency (ANF), and performed classification using random forest classifier. The authors performed experimentation using two benchmark datasets enhancers identification and strength prediction. iEnhancer-RF produced 76.18% and 62.53% accuracy, 73.64% and 68.46% sensitivity, 78.71% and 56.61% specificity and 0.5264 and 0.2529 MCC over core datasets of enhancers identification and strength prediction. Similarly, over independent test set of enhancers identification and strength prediction detests it produced 79.75% and 85% accuracy, 78.50% and 93% sensitivity, 81% and 77% specificity and 0.5952 and 0.7091 MCC, respectively.

Yang *et al*. [36] presented adversarial sequence-generation-based framework, namely, iEnhancer-GAN. For feature engineering they used overlapped and non-overlapped strategies to generate word embedding using skip-gram model. The authors performed experimentation using independent test set of enhancers identification and strength prediction. iEnhancer-GAN produced performance values of 78.4%, 81.1%, 75.8% and 0.567, respectively, in terms of accuracy, sensitivity, specificity and MCC on enhancers identification dataset. On the other hand, over strength prediction dataset it produced 74.9% accuracy, 96.1% sensitivity, 53.7% specificity and 0.505 MCC. Kamran *et al*. [16] proposed a deep-learning-based framework, namely, iEnhancer-Deep, that used one-hot encoding scheme to transform DNA raw sequences into statistical vectors and CNN as a classifier for the identification of enhancers and their strengths. The authors performed extensive experimentation using core datasets and independent test set of both tasks enhancers identification and strength prediction. On benchmark core datasets of enhancers identification and strength prediction, it produced 87.77% and 80.86% accuracy, 86.99% and 83.57% sensitivity, 88.54% and 78.16% specificity and 0.75 and 0.62 MCC, respectively. Similarly on independent test sets of enhancers identification and strength prediction datasets it produced 74.02% and 61% accuracy, 81.5% and 73% sensitivity, 67% and 49% specificity and 0.49 and 0.226 MCC, respectively.

Liao *et al*. [52] proposed a deep-learning-based framework iEnhancer-DCLA that extracts the semantic information between k-mers through the word2vec approach. This semantic information is passed to the CNN layer followed by the attention-based LSTM layer. To analyze the effect of the proposed model the authors performed experimentation using benchmark core dataset and independent test set of enhancers identification and strength prediction datasets. Using enhancers identification and strength prediction datasets it produced 83.32% and 83.3% accuracy, 84.18% and 89.27% sensitivity, 82.45% and 77.33% specificity and 0.666 and 0.673 MCC, respectively. Similarly on independent test sets of enhancers identification and strength prediction datasets it produced 78.25% and 78% accuracy, 78% and 87% sensitivity, 78.5% and 69% specificity and 0.565 and 0.569 MCC, respectively.

Luo *et al*. [47] presented a transfer-learning-based framework iEnhancer-BERT by utilizing a pre-trained language model DNABERT fine-tuned on enhancers sequences. The authors performed experimentation on two benchmark datasets enhancers identification and strength prediction, and evaluated the proposed framework in terms of three evaluation measures: accuracy, MCC and AUC. Over core datasets of enhancers identification and strength prediction iEnhancer-BERT produced 79.4% and 65.3% accuracy, 0.593 and 0.31 MCC and 87.9% and 70.3% AUC, respectively. Similarly over independent test sets of enhancers identification and strength prediction datasets it produced 79.3% and 70.1% accuracy, 0.585 and 0.401 MCC and 84.4% and 81.2% AUC, respectively. Geng *et al*. [51] proposed the RankGAN-based framework in which firstly the authors increased the number of samples through GAN by considering the enhancers dataset small. Secondly, the FastText embedding approach is used to transform the DNA raw sequences into statistical vectors. These vectors passed to the deep learning hybrid model LSTM-CNN that managed to produce 75.25% and 69.7% accuracy, 74.87% and 70.68% sensitivity, 75.63% and 68.89% specificity and 0.5051 and 0.3954 MCC over two core datasets enhancers identification and strength prediction, respectively. Similarly, over independent test sets of enhancers identification and strength prediction it produced 75.25% and 69.7% accuracy, 74.87% and 70.68% sensitivity, 75.63% and 68.89% specificity and 0.5051 and 0.3954 MCC, respectively.

Xiao *et al*. [58] proposed the iEnhancer-MRBF framework that utilizes three different types of sequence encodings, namely, kmer, ANF and nucleotide binary profiles (NBPs). Further, we passed this encoding vector to the Laplacian radial-based classifier RBF. Over benchmark core datasets of enhancers identification and strength prediction, it managed to achieve 81.23% and 76.95% accuracy, 79.52% and 77.23% sensitivity, 82.95% and 79.69% specificity and 0.625 and 0.541 MCC, respectively. Similarly on independent test sets of enhancers identification and strength prediction datasets it achieved 79.75% and 83.50% accuracy, 82% and 100% sensitivity, 77% and 67% specificity and 0.595 and 0.709 MCC, respectively. Li *et al*. [49] presented a BERT-based language model namely iEnhancer-ELM that captures contextual information of k-mers with four different combinations: 3-mer, 4-mer, 5-mer and 6-mer. The authors utilized enhancers identification dataset with two different evaluations: 5-fold cross validation and independent test set. Over independent test iEnhancer-ELM achieved performance values of 83%, 80%, 86% and 0.6612 in terms of accuracy, sensitivity, specificity and MCC, respectively. Over 5-fold cross-validation it achieved performance values of 94.74%, 93.73%, 95.75% and 0.8951 in terms of accuracy, sensitivity, specificity and MCC, respectively.

## MATERIALS AND METHODS

This section describes the working paradigm of the proposed framework along with the benchmark datasets and evaluation metrics used to evaluate the performance of the proposed framework.

### Proposed ADH-enhancer methodology

In NLP and biological sequence analysis tasks AI frameworks’ performance rely on the competence of representation learning methods that transform textual or biological sequence data into statistical feature space [61–64]. Researchers have established a plethora of effective representation learning approaches ranging from different statistical encoders, neural embeddings [37, 65, 66], to language models [67–69]. The prime focus of every other representation learning method is to capture discriminative and semantical relationships of words in NLP and neucelotides in DNA or RNA sequences [2, 66, 70, 71].

Our proposed ADH-Enhancer framework comprises of two major modules, namely, representation learning or sequence transformation to statistical feature space and classification. Representation learning module uses the Universal Language Model Fine-Tuning based optimized language model that is trained in an unsupervised fashion by predicting the next nucleotides based on the previous known neucleotides. Classification module uses a novel classifier that reaps the benefits of two different neural architectures, namely, CNN and attention mechanism. Graphical illustrations of both modules of the proposed ADH-Enhancer framework are given in Figures 2 and 3, and a detailed workflow of each module is discussed in the following subsections.

**Figure 1:**
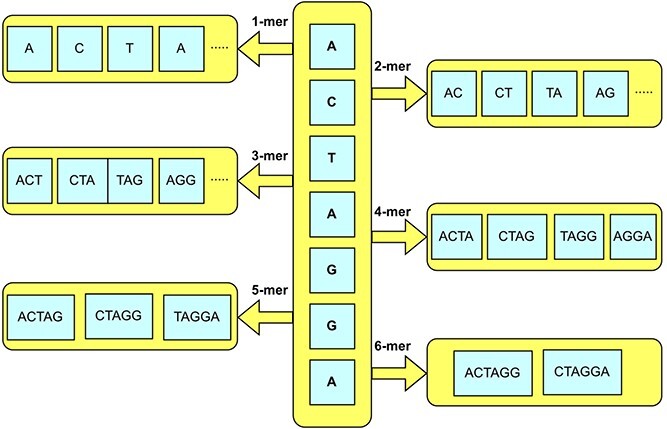
K-mers generation for enhancers sequences.

**Figure 2:**
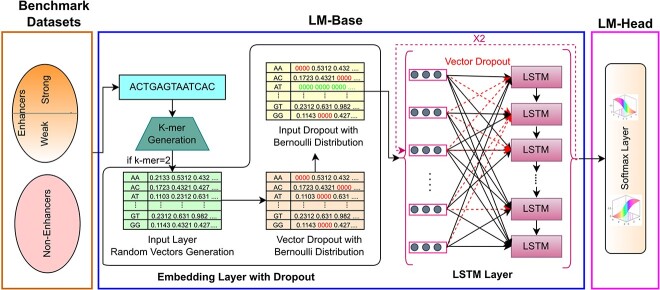
Workflow of self-supervised pre-training and fine-tuning of ULMFIT Module.

#### Statistical representation learning

DNA sequences are comprised of repetitive patterns of four basic nucleotides namely adenine (A) guanine (G) cytosine (C) and thymine (T) [2]. Expanding upon the extensive research in biomedical sequence analysis, it found that discriminative patterns can be extracted when sequences are expressed in terms of combination of basic nucleotides [1]. Here we generate a combination of nucleotides called k-mers of DNA sequences. Figure 1 represents k-mer generation process of a sample sequence ‘ACTAGGA’. Further, these k-mers are passed to neural language model namely ULMFIT. ULMFIT uses self-supervised learning where the model is trained by predicting the next k-mer based on the context provided by the previous k-mers in the sequence. This helps the model in learning contextual relationships between k-mers and their inherent meanings.

**Figure 3:**
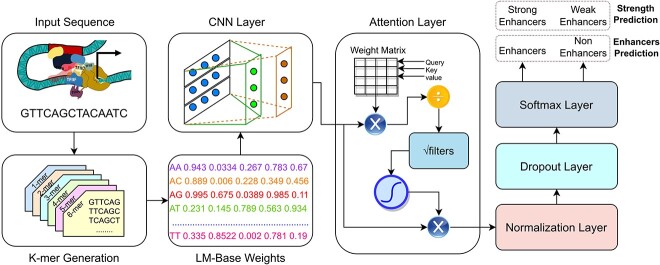
Workflow of attention-based CNN classifier.

ULMFIT is based on a stack of multiple layers including the stochastic embedding layer, the AWD-LSTM layers and the output layer [72], as illustrated in Figure 2.

Stochastic embedding layer converts the input k-mer sequences into numerical representations which can be processed by the model. It maps each k-mer of input sequences to a fixed-size vector and generates an embedding weight matrix $E \in{R}^ {|unique\_kmers| \hspace{0.1cm} \times \hspace{0.1cm} embedding\_size}$. The number of rows in the matrix is equal to the number of unique k-mers, and the number of columns is equal to the size of the k-mer embeddings. To fine-tune the embedding matrix in a more general way, we introduce two different types of dropouts on embedding matrix. The first type of dropout replaces certain k-mer embedding vectors with zeros using the probability of $p_{embeddings}$, while the second type replaces individual continuous values within the remaining k-mer embedding vectors with zeros using the probability of $p_{embeddings\_dim}$. These dropouts help to regularize learnable parameters and prevent model overfitting by ensuring that the model does not over-specialize certain k-mers. The dropout probabilities ($p_{embeddings}$, $p_{embeddings\_dim}$) are tweaked from 0.001 to 0.0008; however the best results are achieved using 0.001. The embedding matrix containing the $400$-dimensional embedding vectors of each k-mer is passed to a stack of three regularized LSTM layers called AWD-LSTM.

AWD-LSTM processes the input sequences sequentially and learn the context and dependencies of k-mers. Unlike the trivial recurrent neural network (RNN), LSTM cellsuse several gates expressed in the following equations to filter the flow of k-mer information. 


(1) \begin{align*} & Input Gate (\bar I_{u}) = sigmoid (W^{i}.x_{t}+U^{i}.h_{t-1} + b_{u}) \end{align*}

(2) \begin{align*} & Forget Gate (\bar I_{f}) = sigmoid (W^{f}.x_{t}+U^{f}.h_{t-1} + b_{f}) \end{align*}

(3) \begin{align*} & Output Gate (\bar I_{o}) = sigmoid (W^{o}.x_{t}+U^{o}.h_{t-1}+ b_{o}) \end{align*}

(4) \begin{align*} & cin_{t} = tanh (W^{c}.x_{t}+U^{c}.h_{t-1}) \end{align*}

(5) \begin{align*} & memory cell state (c_{t}) = (\bar I_{u} \odot cin_{t}+ \bar I_{f} \odot c_{t-1} \end{align*}

(6) \begin{align*} & hidden state (h_{t}) = (\bar I_{o} \odot tanh (c_{t})) \end{align*}

The input, forget and output gates of AWD-LSTM are activated or deactivated based on their weight matrices and biases, and they use activation functions (such as sigmoid or tanh) to determine which information should be retained or discarded from the memory cell states ($c_{t}$, $c_{t-1}$). To preserve k-mer information for an extended time frame, the hidden state $h$ of every cell is conserved at each time step $t$.

To better regularize the recurrent layer, unlike the existing methodologies which randomly drop hidden states while updating the memory state $c_{t}$, AWD-LSTM employs a special dropout technique called DropConnect that applies dropout with a probability of 0.4 to the recurrent weight matrices [$U^{i}$, $U^{f}$, $U^{o}$] as well as non-recurrent weight matrices [$W^{i}$, $W^{f}$, $W^{o}$] of the LSTM layer before the forward and backward passes. This helps the LSTM layers to extract informative features and long-range dependencies more effectively. The AWD-LSTM model in which each LSTM layer uses 256 hidden units is capable of identifying the short- and long-range dependencies of features that are important for enhancer sequence analysis. The resulting 256-dimensional feature vectors are then fed to the output layer.

#### Classifier

With an aim to best utilize context and translational invariance aware statistical representations of k-mers generated by base-language model, we have developed a precise yet robust classifier. Figure 3 illustrates graphical representation of proposed classifier that makes use of convolutional layer to extract comprehensive discriminative features and attention layer to focus on specific features that are more important to distinguish enhancers from non-enhancers as well as strong enhancers from weak enhancers. Attention distribution aware features are passed from normalization and dropout layer before feeding to softmax layer for final prediction. The combination of language model and attention based convolutional layer allow the model to make more informed decisions about the classification of enhancers by considering the context in which k-mers are used and the relative importance of different parts of the sequences. A brief description of different layers of proposed classifier is given in following subsections.

##### Convolutional Layer

The convolutional layer is extensively being used in tasks related to NLP and Bioinformatics because of its local perception and parameter sharing capabilities. It functions like cells in the human brain, using a process called convolution to extract relevant features and simplify the neural network by using shared weights and local connections. The convolution operation at a particular layer $l^{th}$ generates a feature map $A^{[l]}$ which can be represented mathematically as: 


(7) \begin{align*}& A^{[l]}=f(A^{[l]} \quad \otimes W^{[l]} + b^{[l]})\end{align*}

The convolutional kernel weight matrix is represented by $W^{[l]}$ for $l$ layer, symbol $\quad \otimes $ represents convolutional operation, $b^{[l]}$ denotes off-set vector, and f(x) represents activation function. In our experimentation, we have used ReLu activation to mainly sparse the output of convolution layer which leads to accelerate training and promote steady convergence rate by avoiding vanishing gradient problem. CNN layer makes use of 50 kernels having the size of 3 to generate information features based 50-dimensional feature vectors that are fed to the attention layer of proposed neural network.

##### Attention Layer

Attention is a mechanism used in NLP and other fields to weight the importance of different features in a sequence. It is often used in neural networks to allow the model to focus on specific parts of the input when making a prediction. Attention function mainly maps Query vectors (Q), key vectors (K), and Value Vectors to output vectors. Here all three Q, K, V vectors are linear projections of the given enhancer sequences representations and attention function generates new statistical representations of exact same dimensions by incorporating extensive mutual association of k-mer present in enhancer sequences. In our experimentation, we have used scaled dot product as attention function and computed attention in four different steps. First, we calculate the dot product of the query (Q) and key (K) matrices that measure the similarity between the two matrices. The dot product is then scaled by a scaling factor, which is typically the square root of the dimensionality of the key matrix. This scaling is done to prevent the dot product from becoming too large, which can cause issues with numerical stability. Then we apply a softmax function on scaled dot product output to obtain probability distribution. In following equation 8, Weight denotes a square matrix which has number of rows or columns equal to the length of enhancer sequences. 


(8) \begin{align*}& Weight=softmax \frac{QK^{T}}{\sqrt{d_{k}}}\end{align*}

The attention weights are then calculated by taking the dot product of the softmax probabilities with the value matrix (V). The attention weights represent the importance of each element in the value matrix. Finally weighted sum of value matrix is calculated using the attention weights, which is mathematically expressed in equation 9. 


(9) \begin{align*}& y_{i}=\gamma*\hat{x_{i}}+\beta\end{align*}

The weighted sum represents the final attention-weighted output of the scaled dot product attention mechanism. Given 50-dimensional representations of enhancer sequences, scaled dot product attention mechanism modifies the statistical values of features in such a way that features important for enhancer sequence analysis will have better statistical values as compared with those features which are less useful for enhancer sequence analysis.

##### Normalization Layer

A major problem in neural networks is internal co-variance shift, which happens when the distribution of inputs to the hidden layers of the network changes after the model weights are updated during each batch. This can destabilize the neural network and make the optimal weights learned during previous iterations useless, disrupting the convergence and generalizability of the model. To avoid this issues, in proposed classifier, we have used a normalization layer, which involves standardizing the input before it is fed into a hidden layer for each batch. This helps to prevent the input-to-output mapping of the neural network from over-specializing on a specific region of enhancer sequences, leading to faster training, better convergence, and improved generalizability. The process of normalization can be understood using following mathematical expressions and descriptions.

Given a mini-batch of sequences X with m examples and n features, the activations of a layer Z can be computed as $Z = WX + b$, where W is the weight matrix and b is the bias vector. To normalize the activations, we first calculate the mean and variance of the mini-batch: 


(10) \begin{align*} & \mu = \frac{1}{m} \sum_{i=1}^{m} z_{i} \end{align*}

(11) \begin{align*} & \sigma^{2} = \frac{1}{m} \sum_{i=1}^{m} (z_{i} - \mu)^{2} \end{align*}

We can then use these statistics to normalize the activations: 


(12) \begin{align*}& \hat{z_{i}} = \frac{z_{i} - \mu}{\sqrt{\sigma^{2} + \epsilon}}\end{align*}

Where epsilon is a small constant added to the variance to ensure that it is never zero.

Finally, we apply learnable scale and shift parameters gamma and beta to the normalized activations to scale and shift them as needed: 


(13) \begin{align*}& z_{scaled} = \gamma \hat{z_{i}} + \beta\end{align*}

This normalized and scaled version of the activations is then used as input to standard dropout layer of proposed neural network.

##### Dropout Layer

Dropout is a widely used strategy for regularizing neural networks, which helps to avoid over-fitting by arbitrarily dropping a specific percentage of activations in a layer during the training. This forces the network to learn more robust features, prevent hidden unit co-adaptations so that every hidden unit cannot rely on the other hidden units to compensate for their errors.

Given a layer of hidden units A, we apply dropout by setting each activation $A\_i$ to zero with probability $p$, value of which ranges from 0.01 to 0.4. 


(14) \begin{align*}& A_{i} = A_{i} * Bernoulli(1 - p)\end{align*}

Here Bernoulli(p) is a random variable that takes on the value 1 with probability p and 0 with probability 1 - p.

In order to ensure that the expected value of the activation is not changed by the dropout process, we also scale the activations by a factor of 1/(1 - p): 


(15) \begin{align*}& A_{scaled} = A_{i} * 1/(1 - p)\end{align*}

This scaled version of the activations is then used as input to softamx layer of proposed neural network. During test time, we do not apply dropout to the activations. Instead, we use the full set of activations without any dropout applied.

##### Softmax Layer

Using normalized 50 dimensional statistical representations of enhancer sequences, softmax layer distinguishes enhancers from non-enhancers as well strong enhancers from weak enhancers. Softmax can be mathematically expressed using equation 16 where $z$ is the input vector, $i$ is the index of the element in the output vector, and $K$ is the number of classes. The function converts each element in the input vector into a non-negative value between 0 and 1, such that the sum of all the values is 1. This allows the output of the softmax function to be interpreted as a probability distribution over the classes. 


(16) \begin{align*}& \sigma(z)i = \frac{e^{z_{i}}}{\sum{j=1}^{K} e^{z_{j}}}\end{align*}

We use categorical cross-entropy which is often used as the loss function for a neural network classifier with a softmax output layer. It measures the difference between the predicted probability distribution and the true probability distribution for each class. The categorical cross-entropy loss is defined as follows: 


(17) \begin{align*}& L = -\sum_{i=1}^{K} y_{i} \log(\hat{y}_{i})\end{align*}

Where $y$ is the true probability distribution (a vector of length $K$), and $\hat{y}$ is the predicted probability distribution (also a vector of length $K$). The loss is calculated by summing the negative log of the predicted probability for each class.

### Benchmark datasets

**Figure 4:**
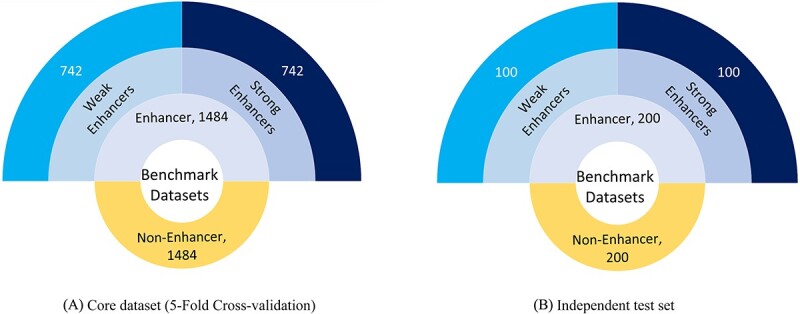
Number of Ssequences in core dataset and independent test for two-Layer prediction involving enhancer identification and its strength prediction.

To evaluate the integrity and generalizability of proposed framework, selection of suitable datasets is an important task [73]. Following the need of enhancer identification and strength prediction datasets, in 2016, Liu *et al*. developed two public benchmark datasets [12]. Specifically, one dataset for enhancer identification and one dataset for strength prediction. Authors also provided independent test sets for both datasets. Almost all the recent frameworks are evaluated on these benchmark datasets. To make sure fair performance comparison of proposed framework with existing frameworks, we also utilize same benchmark datasets. A detailed description about datasets development process is illustrated in Liu *et al* paper [12]. Figure 4 illustrates multi-layered Donut charts that provide statistics of both benchmark data-sets. As shown in the outermost layer, the core dataset has 2,968 DNA sequences in total, among these 50% sequences fall under the hood of enhancer class and rest of the 50% belong to non-enhancer class. A total of 742 sequences constitute each of the 50% enhancer sequences, 25% of which are strong enhancers and the remaining 25% are weak enhancers. In addition to this the central donut chart displays the characteristics of an independent test set which comprises of only 200 enhancer sequences. Among these 200 enhancer sequences, 100 enhancer sequences are strong enhancers while the remaining 100 are weak enhancers.

### Evaluation measures

Following evaluation criteria of existing studies [2, 3, 12, 16], we evaluate our proposed framework using 6 different evaluation measures namely accuracy (Acc), Sensitivity (Sen), Specificity (Spec), Mathhews Correlation Coefficient (MCC), Area Under Receiver Operating Characteristics (AUROC), and Area Under Precsion Recall Curve (AUPRC) 


(18) \begin{align*}& f(x)=\left\{\begin{matrix} ACC= \frac{TP+FP}{TP+FP+TN+FN} \\ SP= \frac{TN}{TN+FP} \\ SN= \frac{TP}{TP+FN} \\ MCC= \frac{(TP*TN)-(FP*FN)}{\sqrt{(TN+FN)* (TN+FP)* (TP+FN)*(TP+FP)}} \\ AUROC= \frac{1}{2} (\frac{TN}{TN+FP} + \frac{TP}{TP+FN}) \\ AUPRC= \frac{1}{2} (\frac{TP}{TP+FN} * \frac{TP}{TP+FP}) \end{matrix}\right.\end{align*}

Equation 18 illustrates mathematical expressions of these measures, number of positive observations and number of negative observations correctly identified in their corresponding classes are represented as true positive ($T_{p}$) and true negative ($T_{N}$), respectively. Whereas the positive observations wrongly predicted as negative observation are represented by false negative ($F_{N}$) and negative observations miss-classified as positive observations are referred by false positive ($F_{p}$). Additionally, MCC, AUROC and AUPRC are utilized to assure that the performance of the proposed ADH-Enhancer framework is not influenced by the size of corpus classes. MCC calculates overall framework’s performance by taking into consideration the size of the positive and negative classes as well as the four performance matrices including $T_{p}$,$T_{n}$,$F_{p}$ and $F_{n}$. AUROC aids in analyzing the trade-off between the true positive rate and false positive rate by giving equal attention to true positives and true negatives. While AUPRC focuses primarily on how effectively the model can predict positive class from all input data, it also examines the trade-off between true positive rate and positive projected value.

## EXPERIMENTAL SETUP

Proposed ADH-Enhancer framework is implemented in python by utilizing 8 different APIs namely; Biopython(https://biopython.org/), Pytorch (https://pytorch.org/), Fastai(https://www.fast.ai/), Pandas (https://pandas.pydata.org/), Plotly(https://plotly.com/), matplotlib (https://matplotlib.org/), numpy (https://numpy.org/) and dash(https://dash.plotly.com/reference). ADH-Enhancer framework comprises of two modules, Base language model and classification head. In base language model we optimized following hyper-parameters, batch size, learning rate, embedding vector dimension. We tweaked batch size value from 16 to 256 with a step size of $2^{n}$ where (n € 4, 5, 6, 7, 8). Similarly, learning rate is tweaked between $e^{-1}$ to $e^{-5}$ with a step size of $e^{-1}$. Embedding vector size is tweaked from 100 to 500 with a step size of 100. For all three hyper-parameters, from given search space to find optimal value of each hyperparameter we employed grid search strategy. Similarly, in classification module, we optimized number of CNN layers, number of filters and kernel size of each CNN layer. We tweaked number of CNN layers from 1,2, and 3. Similarly number of filters on each CNN layer is tweaked from 10-50 range with step size of 10 and kernel size is tweaked from 1-5 with step size of 1. Table 2 illustrates the search space of different hyperparameters and optimal selected values of hyperparameters for language model and classifier. To train the language model and classifier categorical cross entropy loss function and Adam optimizer is used. To reach model convergence early stopping is utilized that stops the model training at the point when loss becomes increases. Furthermore, following evaluation criteria of existing studies [33] [31] [59] [32] [43] [44] [37] [2], we perform experimentation using 5-fold cross-validation and independent test set based settings.

**Table 2:** A comprehensive summary of search space and optimal values of hyper-parameters

Expression	Search Space	Optimal Value		
		Language Model	Classifier	
			Enhancers Identification	Strength Prediction
Number of LSTM layers	1,2,3	3	-	-
Number of neurons	32, 64, 128, 256, 512	256	-	-
Number of CNN layers	1,2,3	-	1	1
Number of filters	10,20,30,40,50	-	30	30
Kernal size	1,2,3,4,5	-	3	3
Weight Decay	$1e^{-1}$ , $1e^{-2}$, $1e^{-3}$, $1e^{-4}$, $1e^{-5}$	$1e^{-2}$	$1e^{-4}$	$5e^{-3}$
Batch size	16, 32, 64, 128, 256	64	64	64
Dropout	$1e^{-1}$ , $1e^{-2}$, $1e^{-3}$, $3e^{-3}$, $5e^{-3}$, $1e^{-4}$, $1e^{-5}$	$1e^{-3}$	$1e^{-2}$	$1e^{-1}$
Embedding size	100, 200, 300, 400, 500	400	400	400
Learning rate	$1e^{-1}$ , $1e^{-2}$, $2e^{-2}$, $3e^{-2}$, $1e^{-3}$, $1e^{-4}$, $1e^{-5}$	$1e^{-4}$	$1e^{-3}$	$2e^{-2}$
Early Stopping	1-200	50	5	7

## RESULTS AND DISCUSSION

This section illustrates the discriminative and distributive potential of nucleotide in enhancer and non-enhancer sequences and further from enhancers in weak and strong enhancers sequences. It also illustrates the performance impact of different size k-mers and statistical representations generated through language model and random embeddings, over two benchmark datasets for tasks in hand. Finally, it presents an in-depth performance comparison of proposed framework with 19 existing frameworks [2, 16, 31–33, 36, 37, 43–45, 47, 49, 51–53, 59, 65, 74, 75].

### Nucleotides distribution analysis

**Figure 5:**
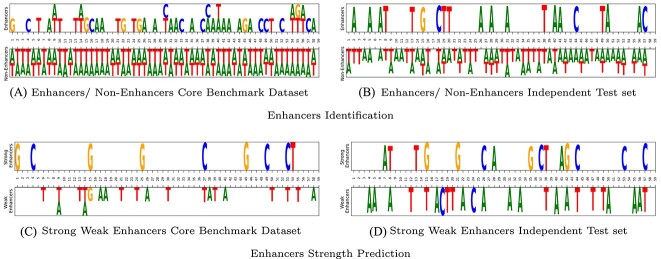
Nucleotides distribution analysis among enhancers identification (Enahncers, Non-Enhancers), and Enhancers Strength Prediction (Weak, Strong Enhancers) using two core benchmark datasets and their independent test sets.

We use sequence logo library [76] to visualize position aware distribution of nucleotides in enhancer and non enhancer as well as weak and strong enhancer sequences. Here aim is to analyze position aware discriminative potential of nucleotides in the sequences of two different classes. Figure 5 represents discriminative potential of four nucleotides (A,C,G,T) in two different classes for two benchmark datasets related to enhancers identification and enhancers strength prediction. Discriminative distribution potential is depicted through the height of character that expresses a nucleotide at each position. To make visualization easy to understandable, we visualize only those nucleotides that have occurrence probability at least 0.75 at relative position.

It can be seen from Figure 5(a,b), in enhancers identification datasets sequences that belong to non-enhancer class have significantly higher prevalence of adenine (A) and thymine (T), while sequences that belong to enhancers class have abundance of guanine (G) and cytosine (C). Similarly, in Figure 5(c,d) visual analysis of enhancers strength prediction datasets shows that sequences that belong to strong enhancers class have higher prevalence of guanine (G) and cytosine (C), and weak enhancers class sequences have these nucleotides in less prevalence. Thus, compositional information of each nucleotide in a sequence has a significance importance because of its discriminative distribution behavior across different classes.

Furthermore, Figure 5 reveals that discriminative behavior shows more prominent when consider consecutive two or three nucleotides. For example in both datasets consecutive ‘AA’, ‘AAA’, ‘TT’ and ‘TTT’ occurs with mess in non-enhancers and weak enhancers class sequences that will facilitate to discriminate the sequences of both benchmark datasets into distinct classes.

### Performance analysis of proposed framework using different k-mers

**Figure 6:**
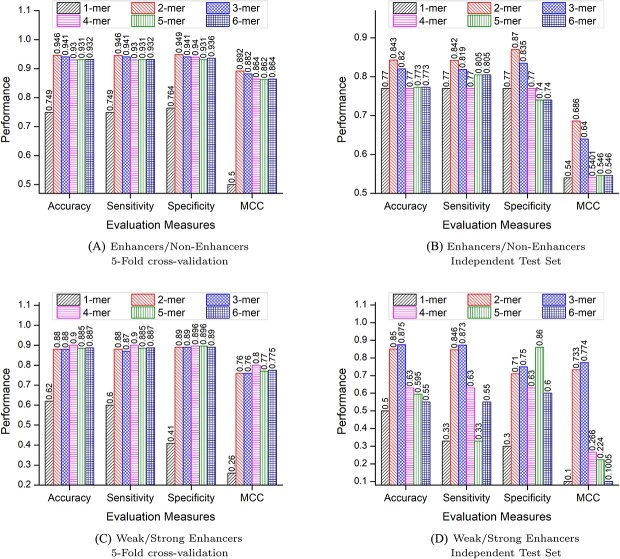
Performance comparison of proposed framework using six different k-mer values over Enhancers identification and strength prediction datasets with two evaluation settings 5-fold cross-validation and independent test set.

Selection of appropriate k-mer size is an important task because the size of k-mer decides unique vocabulary of k-mers and their discriminative potential among sequences of different classes. To find suitable size of k-mer that improve the predictive performance of proposed framework, we perform large scale experimentation by taking n different size k-mers, where $n \varepsilon 1,2,3,4,5,6$. Figure 6 highlights impact of 6 different size k-mers on the predictive performance of proposed framework over two benchmark datasets in-terms of accuracy, sensitivity, specificity and MCC using 5-fold cross validation and independent test set based experimental settings.

Figure 6(a),(b) illustrates that sequences segregated at 1-mer perform worst on enhancers and non-enhancers identification dataset with 5-fold cross-validation and independent test set based experimental settings. Although the performance score of remaining different size k-mers is almost similar but 2-mer of sequences reflect peak performance across all evaluation metrics over 5-fold cross-validation. Furthermore, 2-mers of sequences once again surpasses the performance of remaining different size k-mers over independent test set. In the case of enhancer strength prediction, oscillating performance scores of different size k-mers under both experimental settings can be seen in Figure 6(c),(d). 1-mer of enhancer sequences once again continue to underperform over both experimental settings, which indicates a single residual unit of the sequence is incapable of generating relevant and discriminative features. Enhancer sequences segregated at 3-mer and 4-mer perform best among all other different size k-mers over independent test set and 5-fold cross-validation, respectively. Overall, we observe that 2-mer enables the extraction of more discriminative features of sequences for accurate identification of enhancer and non-enhancer sequences, while for enhancer strength prediction 3-mer and 4-mer extract more discriminative features of sequences.

### Performance impact of language model on enhancers identification and strength prediction

In NLP, transfer learning by training language model in an unsupervised fashion significantly improves the performance of a classifier. Following the success of language models in NLP, our proposed framework predictive pipeline also utilizes the power of language model pre-training. Specifically, to analyze the impact of language model training on the performance of proposed classifier, we perform experimentation in two different experimental settings. In one setting we perform experimentation by using random word embeddings and in other setting, we replace random embeddings with pre-trained language model.

**Figure 7:**
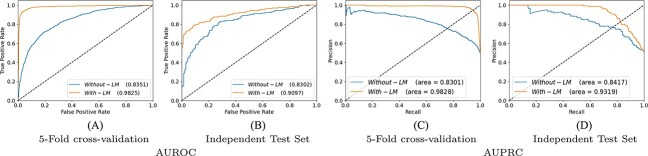
Over enhancers identification dataset with two evaluation settings 5-fold cross-validation and independent test set, performance comparison of proposed framework using language model representations and random embedding ‘without language model’ in terms of AUROC and AUPRC.

**Figure 8:**
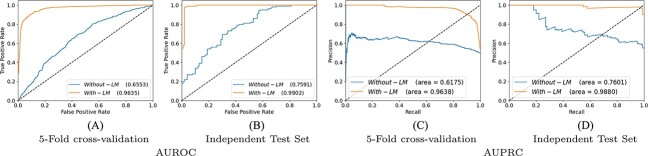
Over enhancers strength prediction dataset with two evaluation settings 5-fold cross validation and independent test set, performance comparison of proposed framework using language model representations and random embedding ‘without language model’ in terms of AUPRC and AUPRC.

Figures 7 and 8 illustrates proposed classifier performance with pre-trained language model and random embeddings over enhancer identification and strength prediction datasets in terms of 5-fold cross-validation and independent test sets. Figure 7 reveals that over enhancer identification dataset with 5-fold cross validation based experimental setting, in comparison with random embedding’s by using pre-trained language model predictive performance of classifiers boost almost 14% and 15% in terms of AUROC and AUPRC. Similarly, over independent test set pre-trained language model improves almost 7% and 9% performance in terms of AUROC and AUPRC. In case of enhancer strength prediction dataset, a similar performance trend can be observed from Figure 8. In 5-fold cross validation based experimental setting, proposed classifier produce 30% and 34% more AUROC and AUPRC performance figures with pre-trained language model as compared with its performance with random word embeddings. Over independent test set proposed classifier in combination with pre-trained language model produce 23% and 22% more performance in terms of AUROC and AUPPRC.

Overall, it can be concluded, pretrained language model significantly improve the performance of classifier because during language model pre-training LSTM layers extract and learn comprehensive information about distribution of nucleotides or k-mers in the sequences.

### Proposed and existing frameworks performance comparison

This section summarizes performance comparison of proposed and 19 existing frameworks for the tasks of enhancer identification and strength prediction using 5-fold cross-validation and independent test set over benchmark datasets. Table 3 illustrates that over 5-fold cross-validation among 19 existing frameworks for enhancer and non-enhancer discrimination, iEnhancer-Deep [16] framework stands out in terms of accuracy and sensitivity with performance values of 87.77% and 86.99% while iEnhancer-CNN [65] framework performs more effectively in terms of specificity and MCC by achieving performance figures of 88.88% and 0.69%, respectively. However, over independent test set iEnhancer-MRBF framework [53] manages to beat the performance of all existing frameworks with 79.75% accuracy, 82% sensitivity and 0.59% MCC while iEnhancer-EBLSTM [74] framework only outperforms existing frameworks in terms of specificity with a value of 79.5%. Hence, existing frameworks are unable to generalize for distinguishing between enhancer and non-enhancer sequences and predicting the strength of enhancers. In contrast to this, proposed framework is capable of extracting discriminative features and outperforms existing top-performing frameworks [16] [53] with a significant margin of 14% and 9% in terms of MCC over 5-fold cross-validation and independent test set respectively.

**Table 3:** Over enhancers identification dataset and its independent test set performance comparison of the proposed and existing frameworks in terms of four evaluation measures

		5-Fold					Independent			
	framework	ACC	Sn	Sp	MCC		ACC	Sn	Sp	MCC
Enhancers/ Non-Enhancer	iEnhancer-2L [33]	76.89	78.09	75.88	0.54		73	71	75	0.46
	EnhancerPred [31]	73.18	72.57	73.79	0.43		74	73.5	74.5	0.48
	tan2019ensemble [44]	74.83	73.25	76.42	0.49		-	-	-	-
	iEnhancer-DSNet [2]	76	76	76	0.52		78	78	77	0.56
	iEnhancer-Deep [16]	87.77	86.99	88.54	0.75		74.02	81.5	67	0.4902
	iEnhancer-EL [43]	78.03	75.67	80.39	0.56		74.75	71	78.5	0.496
	Rank-GAN [51]	75.25	74.87	75.63	0.5051		75.25	74.87	75.63	0.5051
	BERT [48]	76.2	79.5	73	0.525		75.6	80	71.2	0.514
	iEnhancer-XG [45]	58.55	75.7	86.5	0.62		75.75	74	77.5	0.514
	Tan *et al*. Enhancer [44]	74	73	76	0.5		76	76	76	0.51
	iEnhancer-ECNN [75]	-	-	-	-		76.9	78.5	75.2	0.537
	iEnhancer-EBLSTM [74]	-	-	-	-		77.2	75.5	79.5	0.534
	iEnhancer-CNN [65]	80.63	75.88	88.88	0.69		77.5	78.25	79	0.585
	iEnhancer-DCLA [52]	83.32	84.18	82.45	0.6668		78.25	78	78.5	0.565
	iEnhancer-GAN [36]	-	-	-	-		78.4	81.1	75.8	0.567
	Enhancer-RD [36]	-	-	-	-		78.8	81	76.5	0.576
	iEnhancer-5Step [37]	82.3	81.1	83.5	0.65		79	82	76	0.58
	iEnhacner-BERT [47]	79.4	-	-	0.593		79.3	-	-	0.585
	iEnhancer-MRBF [53]	81.23	79.52	82.95	0.6254		79.75	82.00	77.50	0.5956
	Proposed	94.6	**94.6**	94.9	0.892		**84.3**	**84.2**	**87**	**0.686**

With respect to robustness, existing and proposed frameworks fall into two categories: highly biased and less biased frameworks based on differences in specificity and sensitivity values. Highly biased frameworks have higher differences and less-biased frameworks have less difference between sensitivity and specificity values. It is evident from Table 3 that a larger number of frameworks show higher specificity and sensitivity difference under an independent test set in contrast to 5-fold cross-validation. Based on specificity and sensitivity differences (greater than 5), seven existing frameworks including iEnhancer-Deep [16], iEnhancer-EL [43], BERT [48], iEnhancer-GAN [36], iEnhancer-RD [36], iEnhancer-5step [37] and iEnhancer-MRBF [53] are highly biased on independent test set. In contrast to this, only three existing frameworks, namely, Bert [48], iEnhancer-CNN [65] and iEnhancer-XG [45] belong to the highly biased category over 5-fold cross-validation. However, the proposed framework is less-biased as it shows very small specificity and sensitivity difference (less than 5) under 5-fold cross-validation and independent test set. In general, highly biased techniques are prone either toward Type 1 error or Type 2 error. An error of Type 1 occurs when a technique has higher sensitivity and lower specificity values due to a greater number of false positive predictions and an error of Type 2 occurs when a technique has lower sensitivity and higher specificity values due to large number of false negative predictions. Over independent test set except iEnhancer-EL [43] all highly biased frameworks are prone to Type 1 error, whereas over 5-fold cross validation among the three highly biased frameworks two frameworks namely iEnhancer-XG [45] and iEnhancer-CNN [65] are prone to type 2 error.

Table 4 provides a fair performance analysis of the proposed and the 19 existing enhancers strength prediction frameworks under two different experimental settings. It can be seen from Table 4 that among the existing frameworks the iEnhancer-DCLA [52] and iEnhancer-MRBF [53] frameworks produce the highest accuracy, specificity and MCC values over 5-fold cross-validation and independent test set, respectively. On the other hand, iEnhancer-Deep secures the highest performance in terms of sensitivity over 5-fold cross-validation. Comparative performance analysis depicts that the proposed framework outperforms all the existing frameworks over both the experimental settings in terms of the four evaluation measures except for specificity over independent test, where the iEnhancer-CNN framework [65] attains a slightly better performance. Taking into account a higher value of specificity and lower value of sensitivity of the iEnhancer-CNN framework [65] depicts it is highly biased toward type-2 error.

**Table 4:** Over Enhancers strength prediction dataset and its independent test set performance comparison of Proposed and existing frameworks in terms of 4 evaluation measures

		5-Fold					Indepencent			
	framework	ACC	Sn	Sp	MCC		ACC	Sn	Sp	MCC
Weak/ Strong Enhancer	EnhancerPred [31]	62.06	62. 67	61.46	0.24		55	45	65	0.1021
	iEnhancer-2L [33]	61.93	62.21	61.82	0.24		60.5	47	74	0.2181
	iEnhancer-EL [43]	65.03	69	61.05	0.31		61	54	68	0.2222
	iEnhancer-Deep [16]	80.86	83.57	78.16	0.62		61	73	49	0.2266
	iEnhancer-XG [45]	66.74	74.94	58.55	0.33		63.5	70	57	0.272
	iEnhancer-EBLSTM [74]	-	-	-	-		65.8	81.2	53.6	0.324
	iEnhancer-ECNN [75]	-	-	-	-		67.8	79.1	56.4	0.368
	tan2019ensemble [44]	58.96	79.65	38.28	0.19		-	-	-	-
	Tan *et al*. Enhancer [44]	59	80	38	0.2		68.49	0.83	0.46	0.31
	iEnhancer-DSNet [2]	63	63	67	0.26		83	83	67	0.70
	Rank-GAN [51]	69.7	70.68	68.89	0.3954		69.7	70.68	68.89	0.3954
	iEnhacner-BERT [47]	65.3	-	-	0.31		70.1			0.401
	iEnhancer-5Step [37]	68.1	75.53	60.8	0.37		-	-	-	-
	Enhancer-RD [36]	-	-	-	-		70.5	84	57	0.426
	iEnhancer-GAN [36]	-	-	-	-		74.9	96.1	53.7	0.505
	iEnhancer-CNN [65]	76.43	73.64	76.8	0.45		75	65.25	76.1	0.3232
	iEnhancer-DCLA [52]	83.3	89.27	77.33	0.6736		78	87	69	0.5693
	iEnhancer-MRBF [53]	76.95	77.23	79.69	0.5419		83.50	100.00	67.00	0.7098
	Proposed	**90**	**90**	**89.6**	**0.8**		**87.5**	**87.3**	**75**	**0.774**

Over 5-fold cross-validation a large number of existing frameworks including iEnhancer-EL [43], iEnhancer-Deep [16], iEnhancer-XG [45], tan2019ensemble [44], Tan *et al*. Enhancer [44], iEnhancer-5step [37] and iEnhancer-DCLA [52] exhibit unusual behavior with high sensitivity and specificity difference of 7.5%, 5.41%, 16.39%, 41.37%, 42%, 14.73% and 11.94%, respectively. It is interesting to note that all these frameworks are prone toward type-1 error. Hence, these frameworks depict higher false positive rate and incorrectly classify weak enhancers as strong enhancers. In contrast to these existing frameworks the proposed framework shows a negligible difference of 0.4% between the specificity and sensitivity values and hence is independent of type-1 or type-2 errors.

In case of independent test, all the existing frameworks once more exhibit highly biased behavior due to huge differences between sensitivity and specificity values except rank-GAN framework [51]. Among the 19 existing frameworks once again the 10 frameworks including iEnhancer-Deep [16], iEnhancer-XG [45], iEnhancer-EBLSTM [74], iEnhancer-ECNN [75], Tan *et al*. Enhancer [44], iEnhancer-DSNet [2], iEnhancer-RD [36], iEnhancer-GAN [36], iEnhancer-DCLA [52] and iEnhancer-MRBF [53] frameworks are prone to Type 1 error as they have a higher value of sensitivity and a lower value of specificity. However, comparatively a lower number of frameworks, namely, EnhancerPred [31], iEnhancer-2L [33], iEnhancer-EL [43] and iEnhancer-CNN [65] are prone to Type 2 error due to a smaller value of sensitivity and a larger value of specificity. Although the proposed enhancer strength prediction framework also suffers from type-1 error, it has a relatively lower biasness in comparison with the existing frameworks.

## CONCLUSION

In the marathon of developing a robust and precise AI framework for enhancers identification and their strength prediction, researchers have proposed several machine- and deep-learning-based frameworks. These frameworks transform raw DNA sequences into statistical feature space by utilizing physicochemical properties, k-mer neural embeddings, correlation information of nucleotides and bag-of-words. However, these encoding methods are unable to capture the discriminative patterns and semantic relationships of k-mers in a sequence. To address this challenge, we feed an attention-based CNN with context-aware statistical representation of sequences. Over enhancers identification dataset, the proposed ADH-Enhancer framework outperforms the existing best-performing framework by 7%, 8%, 6% and 14% in terms of accuracy, sensitivity, specificity and MCC, respectively. On the other hand, over benchmark enhancers strength prediction dataset it outperforms the existing best by 7%, 1%, 12% and 13% in terms of accuracy, sensitivity, specificity and MCC, respectively. This research study has opened new frontiers for biomedical researchers to explore the potential of language modeling strategies for generating comprehensive statistical representations of sequence to enhance the performance of a variety of biomedical tasks. We believe predictors performance analysis across cross cell-types provides more useful insights about enhancers. However. to perform such analysis there is a need of benchmark datasets having enhancers annotations along with cell lines information.

**Key Points**

- Irregularities in the strength of enhancers affects gene expression process that initiates diverse types of genetic diseases.
- We propose a robust and precise predictor that takes raw DNA sequences and predicts enhancer sites and their strength.
- The proposed predictor transforms raw DNA sequences into statistical feature space by training a language model on large DNA sequence data in unsupervised fashion.
- On top of the pre-trained language model we designed a novel classifier that reaps the benefits of two different architectures: CNN and attention mechanism.

## Author Biographies

**Faiza Mehmood** is serving as a lecturer in computer science department, University of Engineering and Technology Lahore (Faisalabad campus). She did PhD in Computer Science with specialization in bio-informatics at University of Engineering & Technology, Lahore Pakistan. She has been working as a senior researcher (Team Lead) in Al-Khawarizmi Institute of Computer Science (KICS), University of Engineering and Technology Lahore, Pakistan, since 2019. She has completed several natural language processing, bio-informatics and energy projects. She has published various papers in well-reputed journals.

**Shazia Arshad** is serving as a professor in computer science department, University of Engineering and Technology Lahore. Her research interest includes software engineering and bio-informatics.

**Muhammad Shoaib** is serving as a Dean and professor in computer science department, University of Engineering and Technology Lahore. His research interest includes information retrieval, software metrics and bio-informatics.
